# Neuroadrenergic activation in obstructive sleep apnoea syndrome: a new selected meta-analysis - revisited

**DOI:** 10.1097/HJH.0000000000003045

**Published:** 2024-02-15

**Authors:** Annalisa Biffi, Fosca Quarti-Trevano, Matilde Bonzani, Gino Seravalle, Giovanni Corrao, Giuseppe Mancia, Guido Grassi

**Affiliations:** a Department of Statistics and Quantitative Methods, University Milano-Bicocca; b National Centre for Healthcare Research and Pharmacoepidemiology; c Clinica Medica, Department of Medicine and Surgery, University of Milano-Bicocca; d Policlinico di Monza, Monza and University Milano-Bicocca, Milan, Italy

**Keywords:** heart rate, meta-analysis, obstructive sleep apnoea, sympathetic activity

## Abstract

**Background:**

Neuroadrenegic overdrive occurs in obstructive sleep apnoea syndrome (OSAS). However, the small sample size of the microneurographic studies, heterogeneity of the patients examined, presence of comorbidities, represented major weaknesses not allowing to precisely define the main features of the phenomenon, particularly in nonobese patients.

**Objective:**

This meta-analysis detected 14 microneurographic studies based on muscle sympathetic nerve activity (MSNA) quantification in uncomplicated OSAS of different clinical severity.

**Methods:**

The evaluation was extended to the relationships of MSNA with heart rate, anthropometric and blood pressure values, metabolic variables, apnoea-hypopnea index and oxygen saturation.

**Results:**

MSNA is activated markedly and almost homogeneously between studies, showing a progressive increase from the healthy state to mild, moderate and severe OSAS (46.03, 48.32, 71.84, 69.27 bursts/100 heart beats). Of special interest are the findings that MSNA is significantly related to the apnoea-hypopnea index, a marker of OSAS severity (*r* = 0.55, *P* *=* 0.04) but not to BMI, as it occurs in OSAS associated with obesity, and heart rate is significantly and directly related to MSNA and apnoea-hypopnea index (*r* = 0.68 and *r* = 0.60, respectively *P* = 0.03 and *P* = 0.02), thus representing a surrogate marker of the sympathetic overdrive.

**Conclusion:**

OSAS, even when uncomplicated by other cardiometabolic disease, displays a marked sympathetic activation, reflected by the MSNA and heart rate behaviour, becoming a target of therapeutic interventions aimed at exerting sympathomoderating effects, such as continuous positive airway pressure.

## INTRODUCTION

Evaluation of the behaviour of sympathetic cardiovascular neural outflow in obstructive sleep apnoea syndrome (OSAS) has been based throughout the years on three main methodological approaches, namely assay of circulating venous plasma levels of the adrenergic neurotransmitter norepinephrine, power spectral analysis of the heart rate signal and microneurographic recording of efferent postganglionic muscle sympathetic nerve traffic (MSNA) [1–8]. Although technically more demanding but also more sensitive and reproducible than the other two approaches [9], the latter method has been quite systematically used in the past few years for assessing the patterns of sympathetic neural drive in OSAS, becoming the most precise approach to quantify neuroadrenergic outflow in a large number of studies directly or indirectly focused on OSAS. However, because of the limited sample size of the microneurographic studies performed as well as the potential confounding effects of frequently associated clinical conditions, such as diabetes mellitus, obesity, hypertension, metabolic syndrome, renal insufficiency and congestive heart failure, all characterized by an adrenergic overdrive [9–11], the results were often nonhomogeneous and not thoroughly described, preventing conclusive information on different issues to be drawn.

The present meta-analysis has been planned to obtain information on three specific topics. First, whether sympathetic overactivity is demonstrable in OSAS patients without the concomitant presence of cardiovascular and metabolic factors, which ‘per se’ are characterized by sympathetic activation [9–11] and are highly prevalent in this population.

Second, whether the degree of sympathetic activation mirrors the severity of the OSAS. Third, whether the most common biomarker of sympathetic activity used in the clinical setting, that is heart rate, appropriately reflects MSNA, taken as the gold standard for the assessment of sympathetic activity in OSAS patients as well as for reflecting the severity of the disease [8,9].

## MATERIALS AND METHODS

### Data source and search strategy

A systematic review of the medical literature (MEDLINE) was performed according to the Preferred Reporting Items for Systematic Reviews and Meta-analyses (PRISMA) (Supplemental Table S1) [12]. The search was designed by G.G. and G.C. and carried out by A.B. English language studies published up to September 2020 were selected by using the terms ‘muscle sympathetic nerve activity’ AND ‘obstructive sleep apnea’ AND ‘microneurography’ (Supplemental Table S2). Hand-searching allowed the inclusion of two other eligible studies missed during indexing.

### Study selection and data extraction

We selected eight cross-sectional studies [13–20], three prospective studies [21–23] and three clinical trials [24–26] on the basis of the provided details about the behaviour of MSNA collected via the microneurographic technique in patients with OSAS compared to healthy controls. Title and abstracts were screened by two authors (F.Q.-T. and A.B.) and studies were excluded if they were not published in English language, considered individuals concomitantly affected by cardiometabolic disease, such as hypertension, diabetes mellitus and metabolic syndrome because these conditions are characterized ‘per se’ by an increased MSNA [9–11], a proper MSNA quantification as bursts incidence over time and as bursts incidence corrected for heart rate was not possible and publications composed of case reports, reviews, editorials or letters to the editor. When more than one study enrolled part of the same population, we included the most recent and largest article. Thus, the exclusion of duplicate data has been guaranteed by comparing the studies with useful criteria, such as common author names, because most duplicate publication may have authors in common, same country (like institutions or hospitals), clinical condition and same or partially overlapped recruitment period among studies. The overweight state was not considered in the list of comorbidities (see above) because in about half of the study included in the meta-analysis no information was given about the presence/absence of this condition.

The main variable of interest was resting MSNA, quantified as bursts incidence over time (bursts/min) and corrected for heart rate (bursts/100 heart beats). Additional variables extracted were sex and age, BMI (kg/m^2^) and body weight (kg), clinic SBP and DBP (mmHg) and resting heart rate (beats per minute), biochemical data such as plasma cholesterol (mmol/l), low-density lipoprotein (mmol/l), plasma glucose (mmol/l), left ventricular ejection fraction (percentage values) and polysomnographic variables such as apnoea-hypopnea index (number of events per sleep hour), arousal index (number of arousals per sleep hour) and parameters reflecting blood oxygenation, such as minimum O_2_ saturation (percentage values) and O_2_ saturation (percentage values).

Information about the above-mentioned variables are shown in a summary table (Supplemental Table S3) with further characteristics, as first author, year of publication, type of study design and population, details about MSNA and related data. A further variable was represented in some studies by the severity of OSAS, allowing us to perform subgroup analyses concerning this quantitative variable.

### Data analysis

The mean difference in MSNA values between patients with OSAS compared to healthy individuals was chosen as outcome of interest. To this purpose, we evaluated the mean difference and 95% confidence interval (CI) by comparing absolute changes between MSNA mean values in patients with OSAS and in healthy individuals for each study. Stratified analyses were performed to evaluate the role of the study design (specifically prospective or cross-sectional studies). In the studies in which no control group was available, data were analysed taking into account the impact of OSAS severity on MSNA and the relationships with different markers of the disease seriousness. Random effects were used to calculate the pooled estimate [27]. The χ^2^ test and the Higgins I^2^ index were used to evaluate the presence of heterogeneity [28,29]. Publication bias was analysed through the use of a funnel plot and the Egger's test [30]. Finally, in order to identify to what extent the results were influenced by a single study, an influence analysis was conducted by omitting one study at a time. Pearson linear correlations were realized to investigate the association between MSNA means and the mean values of the above-mentioned variables, weighted for the corresponding sample size. All *P* values less than 0.05 were considered statistically significant. Statistical analyses were executed by A.B. using RevMan Version 5.4 (Nordic Cochrane Center) to calculate pooled summary estimates and generate forest plots. The statistical analysis system software version 9.4 (SAS Institute, Cary, North Carolina, USA) for the linear and correlation analysis and STATA Software Program Version 16.1 (STATA, College Station, Texas, USA) was used to calculate the Egger's test [30].

## RESULTS

### Search results

**FIGURE 1:**
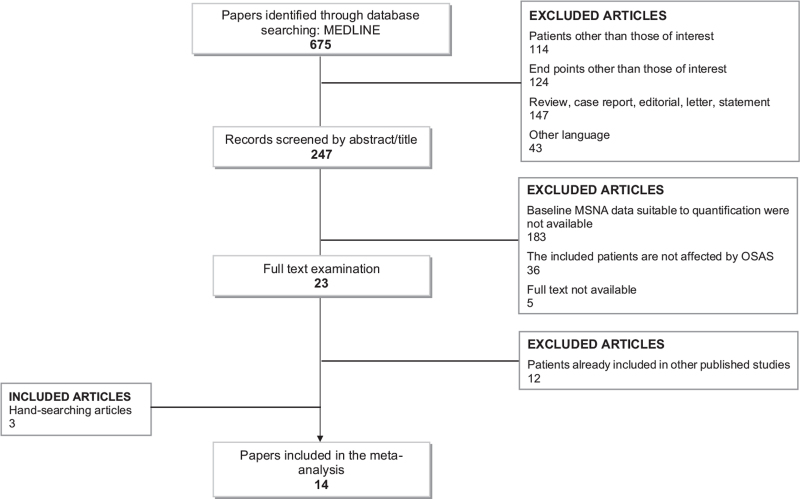
Flow chart of the studies included in the meta-analysis. MSNA, muscle sympathetic nerve activity; OSAS, obstructive sleep apnoea syndrome.

Figure 1 shows the flowchart of the included studies. A total of 675 articles were detected by Medline search, and after title and abstract screening, studies were excluded because considered patients (*n* = 114) or endpoints (*n* = 124) different from those of interest, were reviews, case reports, editorials, letters or statement (*n* = 147), were written in other language (*n* = 43). After full text examination, 183 publications were removed because MSNA data were not available at all or MSNA data expression was lacking either as bursts incidence over time or as bursts incidence corrected for heart rate (see Methods), 36 studies did not include patients affected by OSAS and five full texts were not available. Three additional studies were included by hand-searching and further 12 articles were discarded because of participants were in other published studies. Thus, 14 relevant studies were considered eligible for inclusion in the meta analysis.

### Main studies features

Supplemental Table S3 reports the main characteristics of the eligible articles. The majority of them were conducted in America, few of them in Australia or Japan and just one from Europe. The 14 studies involved 365 patients with OSAS and 119 healthy controls. The 14 studies reported peroneal nerve as site of measurement. In particular, MSNA bursts/100 heart beats was reported for individuals with OSAS (*n* = 270) and healthy controls (*n* = 82) in nine [15–20,23–25] and three [16,17,23] publications, respectively. In addition, MSNA bursts/100 heart beats was investigated depending on the severity of OSAS, specifically mild (78 patients) [16,18–20,24], moderate (58 patients) [18,19,25] and severe (93 patients) [15,16,20,23–25]. Moreover, MSNA expressed as bursts/minute was provided for patients with OSAS (*n* = 223) and healthy individuals (*n* = 74) in 11 [13–16,20–26] and five [13,14,16,22,23] studies, respectively. The quantification of MSNA bursts/minute was tested on the basis of OSAS severity, such as mild (*n* = 37) [16,20,24], moderate (*n* = 15) [25] and severe (*n* = 171) [13–16,21–26].

In general, five studies did not assess patients under pharmacological treatment [17,18,21,24,26], while six articles enrolled individuals without cardiovascular diseases [16–19,25,26], five articles included nonsmokers [17–20,25], two articles enrolled individuals with a history of excessive alcohol consumption [20,24] or individuals who did not use medications for cardiovascular diseases [16,19,25] and one study enrolled individuals who sustained regular physical activity [26] or abstained from caffeine [24]. The majority of the examined studies did not include hypertensive patients [13–19,21,24,26], some of the individuals were overweight/obese [15,17,21,24,26] and without diabetes [17,21,24,26]. Furthermore, some of the selected studies did not provide information on the concomitant presence of diabetes [13,14,16,18,19,22,23] or metabolic syndrome [13,14,16,18,19,22,23].

### Muscle sympathetic nerve activity in patients with obstructive sleep apnoea syndrome versus healthy controls

As specified in the Supplemental Table S4, mean age of individuals with OSAS amounted to 51.6 years compared with 46.2 years of healthy controls. Values of all clinical variables were greater in the OSAS group as compared to those detected in the control one. As expected, the greatest difference was found for AHI variable, among individuals with OSAS (36.2 events per sleep hour), compared with healthy controls (one event per sleep hour). Conversely, O_2_ saturation was higher among healthy controls (98.0%), as compared to patients with OSAS (95.5%). Furthermore, because of the relatively small number of articles available, it was not possible to examine the data according to sex.

**FIGURE 2:**
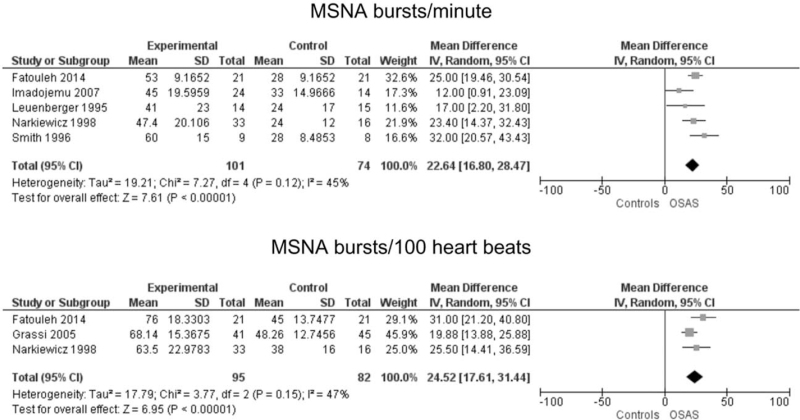
Sympathetic activity in patients with OSAS and healthy controls. Mean difference of muscle sympathetic nerve activity (MSNA) expressed as bursts frequency over time (bursts per minute, upper) and as bursts frequency corrected for heart rate (bursts per 100 heart beats, lower) in patients with OSAS and healthy controls.

**FIGURE 3:**
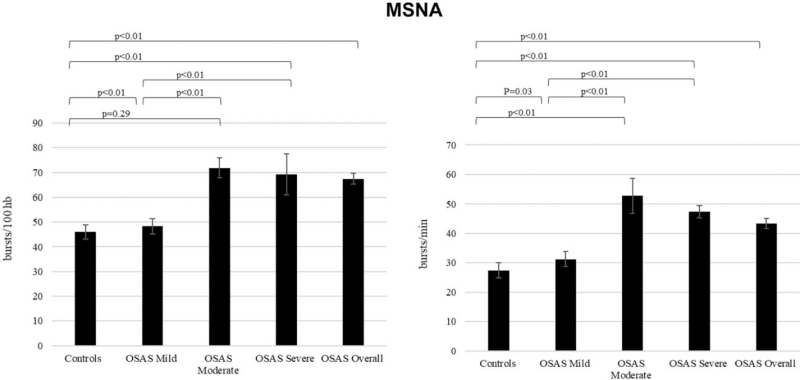
Muscle sympathetic nerve activity expressed as bursts frequency over time (bursts per minute, left) and as bursts frequency corrected for heart rate (bursts per 100 heart beats), right) in healthy controls and in patients with OSAS (specifically mild, moderate or severe). Differences between groups were in the majority of cases statistically significant.

Mean difference of MSNA values, expressed as bursts incidence corrected for heart rate (bursts/100 heart beats), was detected in three studies [16,17,23] between patients with OSAS (*n* = 95) and healthy controls (*n* = 82). The analysis (Fig. 2) shows an increased MSNA bursts incidence corrected for heart rate, 24.52 bursts/100 heart beats (95% CI, 17.61–31.44) with a *P* value less than 0.05. Across the included studies, there was evidence of between-study heterogeneity (*I*^2^ = 47%) and there was not publication bias as detected by funnel plot and confirmed by the *P* value of the Egger's test (Supplemental Figure S1). Moreover, the pooled estimate was not influenced by any of the studies, as verified in the influence analysis (Supplemental Table S5). In addition, five studies [13,14,16,22,23] showed a greater pooled estimate of the mean difference values of MSNA expressed as bursts incidence over time (Fig. 3), which amounted to 22.64 (95% CI, 16.80–28.47, *P* < 0.05) among individuals with OSAS (*n* = 101) compared with healthy controls (*n* = 74). There was no evidence of between-study heterogeneity (*I*^2^ = 45%) nor publication bias as reported by the funnel plot analysis and the Egger's test (Supplemental Figure S1) or influence of any individual study (Supplemental Table S5). Moreover, stratified analyses did not show any role of the study design on the pooled estimates (Supplemental Figure S2).

As shown in Fig. 3, average MSNA values are significantly greater in patients affected by OSAS than in healthy controls, independently of the disease severity, both when MSNA data were expressed as bursts incidence over time (mild OSAS: 31.27 bursts/minute, moderate OSAS 52.70 bursts/minute, severe OSAS 47.40 bursts/minute, overall OSAS: 43.35 bursts/minute, healthy controls: 27.42 bursts/minute) and as bursts incidence corrected for heart rate (mild OSAS: 48.32 bursts/100 heart beats, moderate OSAS: 71.84 bursts/100 heart beats, severe OSAS: 69.27 bursts/100 heart beats, overall OSAS: 67.41 bursts/100 heart beats, healthy controls: 46.03 bursts/100 heart beats).

**FIGURE 4:**
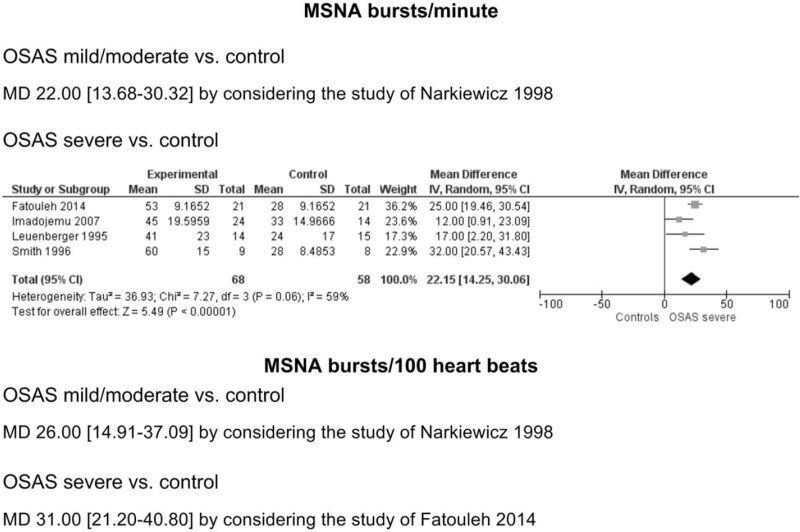
Sympathetic activity in individuals with OSAS (mild/moderate or severe) and healthy controls, expressed as bursts per minute (upper panel) and as bursts corrected for heart rate (bursts/100 heart beats, lower).

Data were also analysed taking into account only the studies providing specific information on severity of the OSAS state. As shown in Fig. 4, which illustrates the data as mean differences between groups, there was a progressive and significant increase in MSNA expressed as bursts incidence over time from healthy individuals to patients with mild-to-moderate OSAS (22.00 bursts/minute, 95% CI, 13.68–30.32) and to those with a severe OSAS state (22.15 bursts/minute, 95% CI, 14.25–30.06) (*P* < 0.01). This was the case also when MSNA data were expressed as bursts incidence corrected for heart rate (Fig. 4, lower), the corresponding values for mild/moderate OSAS patients compared with healthy controls and for severe OSAS compared with healthy individuals being 26.00 bursts/100 heart beats, 95% CI, 14.91–37.09) and 31.00 bursts/100 heart beats, 95% CI, 21.20–40.80).

### Anthropometric, metabolic and haemodynamic correlates of muscle sympathetic nerve activity

**FIGURE 5:**
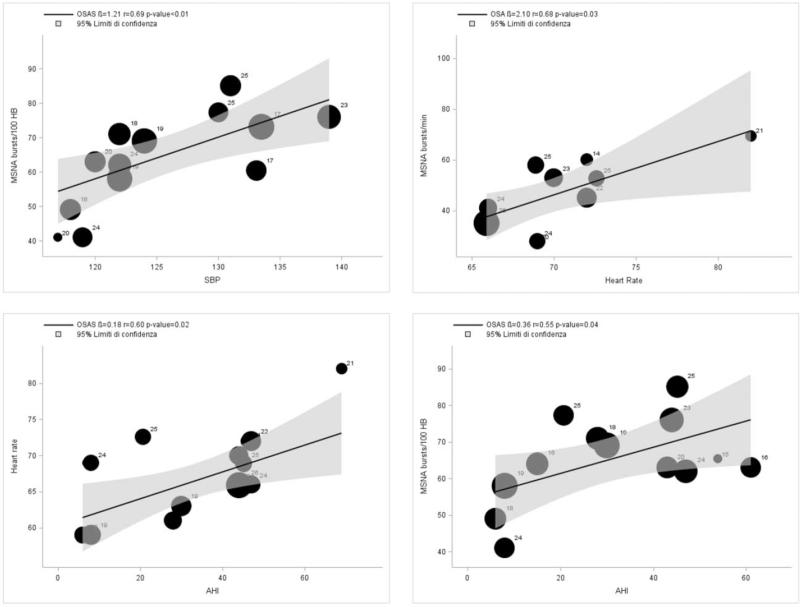
Upper: Regressing SBP (left) and heart rate (right) on muscle sympathetic nerve activity (MSNA), expressed as bursts frequency corrected for heart rate (bursts per 100 heart beats) or bursts per minute in patients with OSAS, with a 95% CI. Lower: Regressing apnoea-hypopnea index (AHI) on muscle sympathetic nerve activity (MSNA, left), expressed as bursts frequency corrected for heart rate (bursts per 100 heart beats) and on heart rate (right) in patients with OSAS, with a 95% CI. Circles represent individual studies (each numbered according to the references) and have a diameter proportional to the sample size of the studies.

Table 1 and Fig. 5 shows the relationships between metabolic, haemodynamic, anthropometric variables and MSNA. In patients with OSAS, MSNA expressed as bursts frequency over time was significantly related to metabolic variables such as plasma glucose (*r* = 1.00, β = 46.81, *P* = 0.04) and LDL cholesterol (*r* = −1.00, β = −69.43, *P* = 0.03), while no significant relationship was detected with BMI. In contrast, MSNA expressed as bursts frequency, respectively, corrected for heart rate and over time, was significantly related to haemodynamic variables, such as SBP (*r* = 0.69, β = 1.21, *P* < 0.01) and heart rate (*r* = 0.68, β=2.10, *P* = 0.03) (Fig. 5, upper). Both MSNA and heart rate were significantly related to apnoea-hypopnea index (*r* = 0.55, β = 0.36, *P* = 0.04 and *r* = 0.60, β = 0.18, *P* = 0.02) (Fig. 5, lower). No significant relationship between MSNA and any anthropometric or metabolic variable was found in healthy individuals, except for heart rate.

**TABLE 1:** Relationship among muscle sympathetic nerve activity bursts/minute (min) or MSNA bursts/100 heart beats and the most relevant clinical variables: correlation coefficient and *P* value

	MSNA bursts/min		MSNA bursts/100 HB	
	OSAS	Control	OSAS	Control
Age (years)				
Correlation coeff.	0.10	0.43	0.33	0.47
Regression coeff.	0.14	0.19	0.57	0.98
*P*	0.73	0.47	0.21	0.53
BMI (kg/m^2^)				
Correlation coeff.	0.24	0.36	0.15	0.40
Regression coeff.	0.63	0.48	0.55	0.83
*P*	0.45	0.77	0.60	0.74
SBP (mmHg)				
Correlation coeff.	0.90		0.69	0.37
Regression coeff.	1.24		1.21	0.48
*P*	**<0.01**		**<0.01**	0.76
DBP *(*mmHg)				
Correlation coeff.	0.42		0.30	0.26
Regression coeff.	1.84		0.60	0.28
*P*	0.31		0.33	0.83
Heart rate (beats/min)				
Correlation coeff.	0.68	–1.00	0.39	0.68
Regression coeff.	2.10	–1.67	0.90	2.01
*P*	**0.03**	**<.01**	0.19	0.52
Cholesterol (mmol/l)				
Correlation coeff.	–0.64			
Regression coeff.	–63.61			
*P*	0.56			
LDL (mmol/l)				
Correlation coeff.	–1.00			
Regression coeff.	–69.43			
*P*	**0.03**			
Glucose (mmol/l)				
Correlation coeff.	1.00		–0.45	
Regression coeff.	46.81		–8.80	
*P*	**0.04**		0.55	
Left ventricular ejection fraction (%)				
Correlation coeff.	–0.59		–1.00	
Regression coeff.	–2.14		–10.73	
*P*	0.41		**<0.01**	
Arousal (events per hour)				
Correlation coeff.	0.50		0.80	
Regression coeff.	0.94		1.24	
*P*	0.50		**0.05**	
Minimum O_2_ saturation (%)				
Correlation coeff.	–0.97		–0.64	
Regression coeff.	–0.87		–1.32	
*P*	**0.03**		0.08	
O_2_ saturation (%)					
Correlation coeff.			0.44	
Regression coeff.			2.77	
*P*			0.38	
Apnoea-hypopnea index (events per hour)				
Correlation coeff.	0.34		0.55	
Regression coeff.	0.20		0.36	
*P*	0.23		**0.04**	

For each variable, data are shown as correlation coefficient, regression coefficient and *P* value. old indicates statistically significant values. BP, blood pressure; MSNA, muscle sympathetic nerve traffic; OSAS, obstructive sleep apnoea syndrome.

## DISCUSSION

The present meta-analysis, which detected 14 microneurographic studies enrolling a total of 484 subjects, represents the largest and more complete evaluation ever done on the behaviour MSNA in OSAS. The main findings can be summarized as follows. First, compared with healthy control subjects without any anamnestic or instrumental evidence of OSAS, patients with documented OSAS display a marked and significant increase in MSNA, both when expressed as bursts frequency over time and as bursts frequency corrected for heart rate. Second, the sympathetic activation in OSAS is detectable in absence of comorbidities known to be characterized by an adrenergic overdrive, such as hypertension, diabetes mellitus, obesity, metabolic syndrome, renal insufficiency and congestive heart failure [9–11,31,32]. Third, the increase in MSNA observed in OSAS is detectable not only in the clinical state as characterized by a marked degree of severity but also in the mild and moderate condition, indicating that the sympathetic activation is an early phenomenon in the clinical course of OSAS and parallels its critical clinical stages.

Several other findings of the present study deserve to be mentioned. The first finding refers to the different relationship between MSNA and anthropometric parameters seen in OSAS patients with or without a concomitant obese state. Indeed, in a meta-analysis recently performed by our group designed to examine the behaviour and determinants of the sympathetic nerve traffic overdrive in the obese state we found that when OSAS is superimposed to an obese state the increase in MSNA appears to maintain a close significant relationship with body weight and body mass index [33]. Surprisingly in the present meta-analysis, the increase in MSNA detected in normoweight or only mild overweight individuals with OSAS does not appear to be significantly associated with body weight or BMI values. This finding suggests that the MSNA increase detected in lean or in just overweight OSAS patients appears to be independent on body weight and it reinforces the notion that OSAS ‘per se’ represents an important determinant of the sympathetic overdrive in this condition via the well known chemoreceptors activation [22]. Second, in the present meta-analysis, we found that in OSAS patients there was a significant direct correlation between MSNA and heart rate, taken as a marker of sympathetic cardiac drive. Third, confirming in a larger population sample the findings by Taylor *et al*. [18], in the present meta-analysis, we saw a significant robust relationship between MSNA, heart rate and the apnoea-hypopnea index. This finding supports the concept that in some clinical conditions, such as OSAS, heart rate may represent a valuable marker of cardiac sympathetic overdrive. It should be emphasized, however, that this appears again different from what we and other investigators found in healthy controls [34], hypertensive patients [35] and uncomplicated obese individuals [33], in which neither heart rate nor plasma norepinephrine values were able to reflect the behaviour of MSNA. Finally, the present meta-analysis shows that in uncomplicated OSAS MSNA values significantly and directly relate to SBP, similarly to what it has been reported in other conditions characterized by sympathetic overdrive, such as obesity, congestive heart failure and hypertension [31–33,36]. This finding strengthens the concept that, independently on the main features of the clinical condition evaluated, a close relationship between MSNA and blood pressure is detectable even when the values of this hemodynamic variable are still in the normal range.

Our meta-analysis has some limitations but also a clinical implication. The limitations concern the fact that not infrequently the design was different between studies included in the analysis to support the information by making more precise estimates although potentially more biased, some reports failed to include in the study population healthy control subjects without OSAS, the OSAS phenotyping was not always well demonstrated, considering that only in a limited number of studies diagnosis was based on polysomnography, the majority of the investigations did not provide information on plasma norepinephrine, preventing us to compare in uncomplicated OSAS the value as sympathetic marker of this adrenergic neurotransmitter vis-a-vis MSNA, as we did in other clinical conditions also characterized by a profound sympathetic overdrive [11,31–33] and there could be a high level of heterogeneity among the considered OSAS and control groups. Potential duplicate publications were discarded to avoid an overestimation of those redundant studies that showed the same nature and direction of the results. Therefore, their exclusion ensured a more reliable summary of studies.

Furthermore, our analysis was not based on individual participant data collected from the included studies; consequently, the findings may be affected by aggregation or ecological bias, that is the causal association at an individual level. This bias is caused by some confounders which are differently distributed among studies (and associated with the aggregate exposure) and related to the outcome [37]. The clinical implication is represented by the importance in OSAS of therapeutic interventions, such as continuous positive airway pressure, capable to counteract the sympathetic overdrive and its adverse structural and functional consequences on the cardiovascular system and the cardiometabolic profile [9–10,21,22].

## ACKNOWLEDGEMENTS

This study revisits the meta-analysis of a prior version [38] and represents a reanalysis of the studies available [39].

### Conflicts of interest

None.

## Supplementary Material

Supplemental Digital Content
