# Supplementary material for: Neuroadrenergic activation in obstructive sleep apnoea syndrome: a new selected meta-analysis - revisited
Source: J Hypertens. 2024 Feb 15;40(1):15–23. doi: 10.1097/HJH.0000000000003045 (PMC10871617; doi:10.1097/HJH.0000000000003045)
Supplement: Supplemental Digital Content [file jhype-40-15-s001.docx]

bursts/min

**
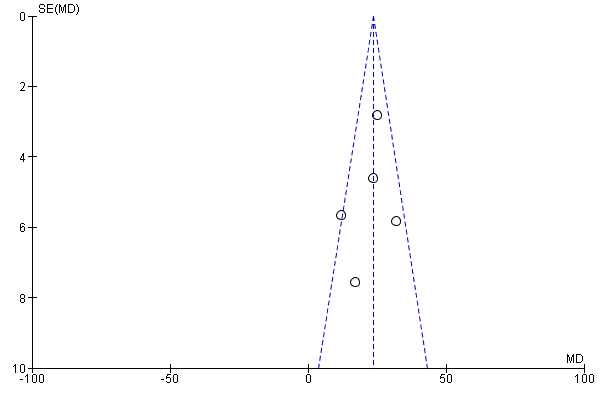
**

Test di Egger: p-value 0.543

bursts/100hb


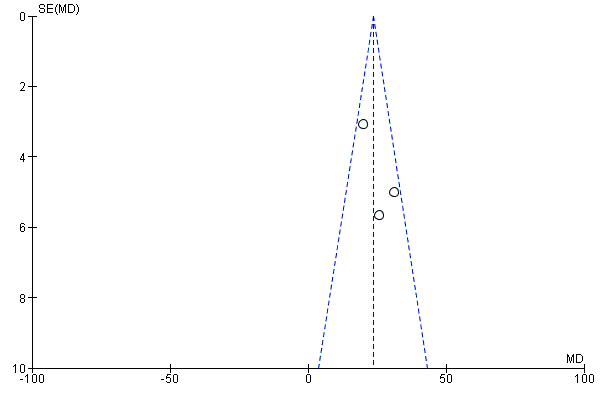


Test di Egger: p-value 0.364

**Supplemental Figure S1.** Funnel plots of MSNA data

Upper panel: subjects with OSAS vs healthy controls for MSNA bursts/min

Lower panel: subjects with OSAS vs healthy controls for MSNA bursts/100hb
