# Supplementary material for: Neuroadrenergic activation in obstructive sleep apnoea syndrome: a new selected meta-analysis - revisited
Source: J Hypertens. 2024 Feb 15;40(1):15–23. doi: 10.1097/HJH.0000000000003045 (PMC10871617; doi:10.1097/HJH.0000000000003045)
Supplement: Supplemental Digital Content [file jhype-40-15-s002.docx]

bursts/min

cross sectional studies


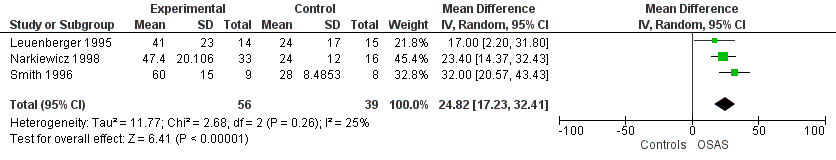


prospective studies

MD 19.42 [6.81, 32.03] by considering the studies of Fatouleh 2014 and Imadojemu 2007.

bursts/100hb

cross sectional studies

MD 21.15 [15.88, 26.43] by considering the studies of Grassi 2005 and Narkiewicz 1998.

prospective studies

MD 31.00 [21.20, 40.80] by considering the study of Fatouleh 2014.

**Supplemental Figure S3.**Sympathetic activity in subjects with OSAS and healthy controls. Mean difference of muscle sympathetic nerve activity expressed as bursts incidence over time (bursts/min, top panel) and as bursts corrected for heart rate (bursts/100 heart beats, bottom panel) in individuals with OSAS and healthy controls evaluated in cross sectional and prospective studies. HB, heart beats.
