# Supplementary material for: Neuroadrenergic activation in obstructive sleep apnoea syndrome: a new selected meta-analysis - revisited
Source: J Hypertens. 2024 Feb 15;40(1):15–23. doi: 10.1097/HJH.0000000000003045 (PMC10871617; doi:10.1097/HJH.0000000000003045)
Supplement: Supplemental Digital Content [file jhype-40-15-s004.docx]

| #1  “Sympathetic activation” OR “sympathetic nerve traffic” OR “sympathetic hyperactivity” OR “sympathetic nerve hyperactivity” OR “sympathetic neural hyperactivity” OR “sympathetic nervous system activity” OR “sympathetic nerve activity” OR “sympathetic neural activity” OR “muscle sympathetic nerve activity” OR MSNA OR microneurograph* OR echocardiographi* OR “sympathetic nerve firing” OR “nerve signal” OR “neural signal” OR microelectrode* OR “bursts/min” OR “bursts/100” OR “burst incidence” OR “burst frequency” OR neurogram* OR “sympathetic nerve discharge” OR “sympathetic nerve discharges” OR “sympathetic neural discharge” OR “sympathetic neural discharges” OR “peroneal nerve” OR “fibular nerve” OR “tibial nerve” OR “peripheral nerve” OR “bursts minute” OR burst*  #2  "Sleep Apnea, Obstructive"[Mesh] OR OSAS[Text word] OR OSAS[All fields] OR "obstructive sleep apnea"[Text word] OR "obstructive sleep apnea"[All fields] OR "obstructive sleep apnoea"[Text word] OR "obstructive sleep apnoea"[All fields] OR "sleep apnea"[Text word] OR "sleep apnea"[All fields] OR "sleep apnoea"[Text word] OR "sleep apnoea"[All fields]OR "sleep apnea-hypopnea"[Text word] OR "sleep apnea-hypopnea"[All fields] OR "sleep apnoea-hypopnea"[Text word] OR "sleep apnoea-hypopnea"[All fields] OR OSAHS[Text word] OR OSAHS[All fields] OR "apnea-hypopnea"[Text word] OR "apnea-hypopnea"[All fields] OR "apnoea-hypopnea"[Text word] OR "apnoea-hypopnea"[All fields] OR "sleep disordered breathing"[Text word] OR "sleep disordered breathing"[All fields] OR "Obstructive Sleep-disordered Breathing"[Text word] OR "Obstructive Sleep-disordered Breathing"[All fields]  #3  #1 OR #2  limit to humans |
| --- |

**Supplemental Table S2.** Focused search strategy in MEDLINE data base.
