# Supplementary material for: Neuroadrenergic activation in obstructive sleep apnoea syndrome: a new selected meta-analysis - revisited
Source: J Hypertens. 2024 Feb 15;40(1):15–23. doi: 10.1097/HJH.0000000000003045 (PMC10871617; doi:10.1097/HJH.0000000000003045)
Supplement: Supplemental Digital Content [file jhype-40-15-s005.docx]

**Supplemental Table S3**. Summary of main characteristics and findings of the studies reviewed.

| **First author, year, country** | **Study design** | **Population**  **Sample size, characteristics** | **MSNA technique** | **MSNA (bursts/min or bursts/100 HB)** |
| --- | --- | --- | --- | --- |
| Fatouleh 2014, Australia | Prospective | OSA SEVERE subjects  Male/Female: 18/3  Age (yrs): 55 ± 2  SBP (mmHg): 139 ± 4  DBP (mmHg): 80 ± 2  AHI events/h: 44  HR: 70 ± 3  Controls  Male/Female: 17/4  Age (yrs): 52 ± 2  SBP (mmHg): 120 ± 3  DBP (mmHg): 68 ± 3  HR: 66 ± 3 | Peroneal Nerve | MSNA burst/100hb  76 ± 4  MSNA burst/min  53 ± 2  MSNA burst/100hb  45 ± 3  MSNA burst/min  28 ± 2 |
| Goya 2016, Brazil | Clinical Trial | OSA SEVERE subjects  Male/Female: 11/9  Age (yrs): 54 ± 2  SBP (mmHg): 122 ± 3  DBP (mmHg): 79 ± 1  AHI events/h: 47  HR: 66 ± 2  BMI: 30  Min O_2_ saturation: 76  Chol mmol/L: 5.21  Glucose mmol/L: 5.77  LVEF %: 69  OSA MILD subjects  Male/Female: 8/7  Age (yrs): 51 ± 2  SBP (mmHg): 119 ± 3  DBP (mmHg): 78 ± 2  AHI events/h: 8  HR: 69 ± 2  BMI: 27  Min O_2_ saturation: 90  Chol mmol/L: 5.28  Glucose mmol/L: 5.49  LVEF %: 71 | Peroneal Nerve | MSNA burst/100hb  62 ± 4  MSNA burst/min  41 ± 3  MSNA burst/100hb  41 ± 3  MSNA burst/min  28 ± 2 |
| Grassi 2005, Italy | Cross Sectional | OSA lean subjects  Male/Female: 13/3  Age (yrs): 47.4 ± 1.8  SBP (mmHg): 133.1 ± 2.3  DBP (mmHg): 84.4 ± 1.1  HR: 70.2 ± 1.4  BMI: 24.3  Glucose mmol/L: 4.31  O_2_ saturation: 97.8  HOMA-IR: 1.79  Waist hip ratio: 0.77  OSA obese subjects  Male/Female: 20/5  Age (yrs): 48.7 ± 1.5  SBP (mmHg): 133.5 ± 2.0  DBP (mmHg): 84.7 ± 1.3  HR: 74.1 ± 1.3  BMI: 32.6  Glucose mmol/L: 4.67  O_2_ saturation: 97.3  HOMA-IR: 3.32  Waist hip ratio: 0.96  Control lean subjects  Male/Female: 21/6  Age (yrs): 47.6 ± 1.6  SBP (mmHg): 131.3 ± 1.9  DBP (mmHg): 82.9 ± 2.2  HR: 69.4 ± 1.3  BMI: 23.9  Glucose mmol/L: 4.15  O_2_ saturation: 98.9  HOMA-IR: 1.54  Waist hip ratio: 0.76  Control obese subjects  Male/Female: 14/4  Age (yrs): 49.1 ± 2.1  SBP (mmHg): 133.8 ± 2.1  DBP (mmHg): 84.0 ± 1.3  HR: 72.7 ± 1.4  BMI: 32.2  Glucose mmol/L: 4.51  O_2_ saturation: 98.1  HOMA-IR: 2.76  Waist hip ratio: 0.95 | They were put in the supine position and fitted with the intravenous cannula, microelectrodes for MSNA recording, and other measuring devices. | MSNA burst/100hb  60.4 ± 2.3  MSNA burst/100hb  73.1 ± 2.5  MSNA burst/100hb  40.9 ± 1.8  MSNA burst/100hb  59.3 ± 2 |
| Guerra 2019, Brazil | Clinical Trial | OSA SEVERE subjects  Male/Female: 24/17  Age (yrs): 51.5 ± 1.5  SBP (mmHg): 120.5 ± 2.5  DBP (mmHg): 78 ± 1.5  AHI: 44  HR: 65.9 ± 2  Weight kg: 81.5  BMI: 29.5  Chol mmol/L: 5.31  LDL mmol/L: 3.38  Glucose mmol/L: 5.66  Arousal events/h: 31  Min O_2_ saturation: 80.1  LVEF %: 67.9 | Peroneal Nerve | MSNA burst/min  35 ± 2.5 |
| Hamaoka 2018, Japan | Clinical Trial | OSA MODERATE subjects  Male/Female: 11/4  Age (yrs): 61.3 ± 3.2  SBP (mmHg): 130 ± 3.8  DBP (mmHg): 81.6 ± 3  AHI: 20.7  HR: 72.6 ± 2.97  BMI: 26.3  LDL mmol/L: 3.11  Arousal events/h: 25.6  OSA SEVERE subjects  Male/Female: 15/2  Age (yrs): 65 ± 2.2  SBP (mmHg): 131 ± 2.8  DBP (mmHg): 76.2 ± 2.9  AHI: 45.2  HR: 68.9 ± 1.89  BMI: 26.2  LDL mmol/L: 3.06  Arousal events/h: 40.9 | Peroneal Nerve | MSNA burst/100hb  77.3 ± 3.8  MSNA burst/min  52.7 ± 3  MSNA burst/100hb  85.1 ± 3.4  MSNA burst/min  57.9 ± 2.9 |
| Imadojemu 2007, USA | Prospective | OSA SEVERE subjects  Male/Female: 18/6  Age (yrs): 50 ± 3  AHI: 47  HR: 72 ± 3  BMI: 34  O_2_ saturation: 96  Controls  Male/Female: 10/4  Age (yrs): 54 ± 4  AHI; 1  HR: 63 ± 2  BMI: 32  O_2_ saturation: 97 | Peroneal Nerve | MSNA burst/min  45.4 ± 4  MSNA burst/min  33 ± 4 |
| Leuenberger 1995, USA | Cross Sectional | OSA SEVERE subjects  Male/Female: 14/1  Age (yrs): 45 ± 3.4  AHI: 65  BMI: 37  Controls  Male/Female: 15  Age (yrs): 49 ± 4.1  BMI: 26 | Peroneal Nerve | MSNA burst/min  41 ± 5.9  MSNA burst/min  24 ± 4.4 |
| Narkiewicz 1998, USA | Cross Sectional | OSA MILD subjects  Male/Female: 14/4  Age (yrs): 45 ± 2.4  AHI: 15  OSA SEVERE subjects  Male/Female: 12/3  Age (yrs): 40 ± 2.3  AHI: 61  Controls  Male/Female: 12/4  Age (yrs): 41 ± 2.3 | Peroneal Nerve | MSNA burst/100hb  64 ± 4  MSNA burst/min  46 ± 3  MSNA burst/100hb  63 ± 4  MSNA burst/min  49 ± 4  MSNA burst/100hb  38 ± 4  MSNA burst/min  24 ± 3 |
| Shimizu 1997, Japan | Cross Sectional | OSA SEVERE subjects  Male/Female: 3/0  Age (yrs): 50.7  SBP (mmHg): 127 ± 3  DBP (mmHg): 79 ± 2  AHI: 53.9  BMI: 25.3 | Peroneal Nerve | MSNA burst/100hb  65.4 ± 5.3  MSNA burst/min  35.7 ± 3.6 |
| Smith 1995, USA | Cross Sectional | OSA SEVERE subjects  Male/Female: 8/1  Age (yrs): 34 ± 4  SBP (mmHg): 141 ± 7  DBP (mmHg): 87 ± 4  AHI: 46  HR: 72 ± 4  Weight kg: 107  Controls  Male/Female: 6/2  Age (yrs): 31 ± 3  SBP (mmHg): 132 ± 4  DBP (mmHg): 80 ± 3  HR: 66 ± 3  Weight kg: 98 | Peroneal Nerve | MSNA burst/min  60 ± 5  MSNA burst/min  28 ± 3 |
| Taylor 2016_arousal from, Canada | Cross Sectional | OSA MILD subjects  Male/Female: 14/10  Age (yrs): 59 ± 1  SBP (mmHg): 122 ± 3  DBP (mmHg): 69 ± 2  AHI: 8  HR: 59 ± 2  BMI: 28  Min O_2_ saturation: 88  O_2_ saturation: 95  Arousal events/h: 20  OSA MODERATE subjects  Male/Female: 16/8  Age (yrs): 58 ± 2  SBP (mmHg): 124 ± 3  DBP (mmHg): 72 ± 2  AHI: 30  HR: 63 ± 2  BMI: 30  Min O_2_ saturation: 83  O_2_ saturation: 95  Arousal events/h: 32 | Unipolar tungsten microelectrode inserted percutaneously into a fascicle of the right common fibular nerve | MSNA burst/100hb  58 ± 4  MSNA burst/100hb  69 ± 3 |
| Taylor 2016_association between, Canada | Cross Sectional | OSA MILD subjects  Male/Female: 11/6  Age (yrs): 57 ± 4  SBP (mmHg): 118 ± 3  DBP (mmHg): 67 ± 7  AHI: 6  HR: 59 ± 3  BMI: 27  Min O_2_ saturation: 87  O_2_ saturation: 94  OSA MODERATE subjects  Male/Female: 15/4  Age (yrs): 58 ± 4  SBP (mmHg): 122 ± 2  DBP (mmHg): 70 ± 9  AHI: 28  HR: 61 ± 2  BMI: 30  Min O_2_ saturation: 82  O_2_ saturation: 95 | Unipolar tungsten microelectrode inserted percutaneously into a fascicle of the right common fibular nerve | MSNA burst/100hb  49 ± 4  MSNA burst/100hb  71 ± 4 |
| Ueno-Pardi 2017, Brazil | Cross Sectional | OSA SEVERE subjects  Male/Female: 8/9  Age (yrs): 53 ± 2  SBP (mmHg): 120 ± 3  DBP (mmHg): 78 ± 1  AHI: 43  HR: 66 ± 2  BMI: 29  Min O_2_ saturation: 78  Arousal events/h: 78  LVEF %: 69  OSA MILD subjects  Male/Female: 5/9  Age (yrs): 51 ± 2  SBP (mmHg): 117 ± 4  DBP (mmHg): 77 ± 2  AHI: 7  HR: 69 ± 3  BMI: 27  Min O_2_ saturation: 90  Arousal events/h: 90  LVEF %: 71 | Peroneal Nerve | MSNA burst/100hb  63 ± 4  MSNA burst/100hb  41 ± 3  MSNA burst/min  28 ± 2 |
| Waravdekar 1996, USA | Prospective | OSA SEVERE subjects  Male/Female: 6/1  Age (yrs): 50 ± 4.2  AHI: 69  HR: 82 ± 9  BMI: 38  O_2_ saturation: 94 | Peroneal Nerve | MSNA burst/min  69.4 ± 5.8 |

Data are mean±SEM. Abbreviations: OSA, obstructive sleep apnea; MSNA, muscle sympathetic nerve activity; SBP, systolic blood pressure; DBP, diastolic blood pressure; BMI, body mass index; HR, heart rate; LDL, low density lipoproteins; LVEF, left ventricular ejection fraction; AHI, apnea-hypopnea index; Chol, cholesterol.
