# Supplementary material for: Neuroadrenergic activation in obstructive sleep apnoea syndrome: a new selected meta-analysis - revisited
Source: J Hypertens. 2024 Feb 15;40(1):15–23. doi: 10.1097/HJH.0000000000003045 (PMC10871617; doi:10.1097/HJH.0000000000003045)
Supplement: Supplemental Digital Content [file jhype-40-15-s006.docx]

**Supplemental Table S4.** Mean values (± Standard Deviation) of the demographic, anthropometric and metabolic variables.

|  | **Controls** | **OSAS** |
| --- | --- | --- |
| N | 119 | 365 |
| Age | 46.24 ± 7.86 | 51.65 ± 7.15 |
| Body mass index | 29.42 ± 4.16 | 29.96 ± 4.02 |
| Apnoea-hypopnea index | 1.00 | 36.20 ± 20.50 |
| Heart rate | 67.42 ± 3.72 | 68.22 ± 5.84 |
| Weight | 98.00 | 94.27 ± 18.01 |
| Systolic blood pressure | 129.28 ± 6.27 | 126.14 ± 7.81 |
| Diastolic blood pressure | 78.73 ± 7.35 | 77.49 ± 5.90 |
| Cholesterol | . | 5.27 ± 0.05 |
| Low density lipoprotein | . | 3.18 ± 0.17 |
| Glucose | 4.33 ± 0.25 | 5.18 ± 0.65 |
| Arousal events per hour | . | 28.21 ± 7.20 |
| Minimum O_2_ saturation | . | 83.79 ± 5.21 |
| O2 saturation | 98.00 ± 0.95 | 95.51 ± 1.42 |
| HOMA-IR | 2.15 ± 0.86 | 2.56 ± 1.08 |
| Left ventricular ejection fraction | . | 69.58 ± 1.37 |
| MSNA bursts/100hb | 45.80 ± 9.45 | 63.64 ± 12.28 |
| MSNA bursts/min | 27.40 ± 3.71 | 45.83 ± 12.14 |

MSNA: muscle sympathetic nerve activity. HOMA: homeostatic model assessment

IR: insulin resistance.
