# Supplementary material for: Neuroadrenergic activation in obstructive sleep apnoea syndrome: a new selected meta-analysis - revisited
Source: J Hypertens. 2024 Feb 15;40(1):15–23. doi: 10.1097/HJH.0000000000003045 (PMC10871617; doi:10.1097/HJH.0000000000003045)
Supplement: Supplemental Digital Content [file jhype-40-15-s007.docx]

**Supplemental Table S5**. Influence analysis investigating the summary mean difference of MSNA, expressed as bursts/min (upper) or bursts/100 heart beats (lower), among subjects with OSA pathology compared to control (reference) by omitting one study at time among those contributing the mean difference (MD) estimates.

**MSNA bursts/min**

| **Omitted study**  **(First author, publication year)** | **Summary MD** | **(95% CI)** | | **Heterogeneity** | |
| --- | --- | --- | --- | --- | --- |
|  |  |  |  | **P-value** | **I^2^** |
| Fatouleh 2014 | 21.40 | 12.99 | 29.81 | < 0.0001 | 54.4% |
| Imadojemu 2007 | 24.96 | 20.77 | 29.14 | < 0.0001 | 0.0% |
| Leuenberger 1995 | 23.34 | 16.77 | 29.91 | < 0.0001 | 54.0% |
| Narkiewicz 1998 | 22.15 | 14.25 | 30.06 | < 0.0001 | 58.8% |
| Smith 1996 | 21.02 | 15.18 | 26.86 | < 0.0001 | 37.1% |
| *Combined estimate* | 22.64 | 16.80 | 28.47 | < 0.0001 | 45.0% |

**MSNA bursts/100 heart beats**

| **Omitted study**  **(First author, publication year)** | **Summary MD** | **(95% CI)** | | **Heterogeneity** | |
| --- | --- | --- | --- | --- | --- |
|  |  |  |  | **P-value** | **I^2^** |
| Fatouleh 2014 | 21.15 | 15.88 | 26.43 | < 0.0001 | 0.0% |
| Grassi 2005 | 28.59 | 21.25 | 35.93 | < 0.0001 | 0.0% |
| Narkiewicz 1998 | 24.74 | 13.93 | 35.55 | < 0.0001 | 72.2% |
| *Combined estimate* | 24.52 | 17.61 | 31.44 | < 0.0001 | 47.0% |
